# In Situ Punch–Shear Testing of Polymers

**DOI:** 10.3390/polym17070981

**Published:** 2025-04-04

**Authors:** David Munoz-Paniagua, Ahmed Hammami, Hadi Nazaripoor, Abderrazak Traidia, Jorge Palacios Moreno, Pierre Mertiny

**Affiliations:** 1 Department of Mechanical Engineering, University of Alberta, 9211-116 St, Edmonton, AB T6G 1H9, Canada; munozpan@ualberta.ca (D.M.-P.); ajorge@ualberta.ca (J.P.M.); 2 Mattr Corp, Flexpipe, 3501 54 Ave SE, Calgary, AB T2C 0A9, Canada; ahmed.hammami@mattr.com (A.H.); hadi.nazaripoor@mattr.com (H.N.); 3 Saudi Aramco, Research and Development Center, P.O. Box 62, Dhahran 31311, Saudi Arabia; abderrazak.traidia@aramco.com

**Keywords:** in situ properties, punch–shear testing, tensile testing, thermoplastic polymers, saturation, aging, reversibility

## Abstract

Conventional material aging and testing protocols involve exposing coupon samples to saturation in application fluid(s) at temperature and pressure conditions typically encountered during service, followed by mechanical testing at ambient conditions. This practice can generate misleading results for materials for which fluid ingress is rapidly reversible, most notably at elevated temperatures. A recently developed in situ punch–shear device has been successfully used to establish experimental correlations between the tensile properties (ASTM D638) and shear properties (ASTM D732) of Polyethylene of Raised Temperature (PERT) under dry conditions. It also enabled measurement of shear properties of select polymers while immersed (saturated) in fluids at elevated pressure and temperature. The present work extends the treatment to a suite of commercially available thermoplastic polymers spanning the commodity, engineering, and high-performance polymer grades with varying degrees of hygroscopicity. The objectives of this contribution are three-fold, namely: (i) assess the effect of sample preparation method on measured mechanical properties, (ii) compare the experimentally established correlations between shear and tensile tests for the different class of polymer grades before fluid exposure, and (iii) gauge reversibility of the measured tensile and shear properties after aging in deionized water to saturation at 95 °C. Results indicate that (i) the test coupon preparation method affects the tensile to shear correlation and must be standardized to enable systematic comparison of in situ properties, (ii) individual correlations segregate by polymer family, and (iii) conventional tensile testing after a saturation–dehydration cycle yields optimistic mechanical properties.

## 1. Introduction

Polymer materials offer cost-effective manufacturing options and selective resistance to specific chemicals [1]. Polymers are primarily classified as thermosets or thermoplastic materials. Thermosets are solidified, cured, or hardened into a permanent configuration, typically expedited by heat or ultraviolet light, resulting in irreversible cross-linking of the polymer. On the other hand, thermoplastics soften upon heating into a flowable condition, allowing them to be molded into functional items. They retain their form once cooled below the point of solidification. Thermoplastics can repeatedly be softened by heating and subsequently molded [2]. Thermoplastics offer recyclability, and a notable decrease in production costs can be accomplished using efficient and continuous fabrication processes [3]. Nonetheless, prior studies have demonstrated that the characteristics of polymer materials can fluctuate significantly between their initial dry condition and subsequent aging in specific fluids.

Thermoplastic materials are increasingly used to build piping for fluid transport applications, such as water service and the oil and gas industries. As such, some scientific works have investigated the effects of aging on their properties. For example, Castillo-Montes et al. [4] investigated the effect of sodium hypochlorite solution on the degradation of polyethylene of raised temperature (PERT). The system degraded at concentrations between 25 and 100 ppm. Later, Kong et al. [5] examined the degradation of PERT pipe liners over 4 years of operation in a crude oil collection system. The findings revealed that the liner was swollen by the conveyed medium, leading to a rough surface, increased density, reduced hardness, diminished thermal stability, decreased thermal dimensional stability, and reduced crystallinity, leading to diminished mechanical characteristics. Zha et al. [6] evaluated the degradation of PERT pipes by accelerated tests with an exposure time of up to 10,000 h as well as for pipes in operation in long-term service. Their results revealed that degradation under oxidative conditions possesses time-dependent properties and spatially heterogeneous oxidation profiles. Due to diffusion-limited oxidation, the pipes showed a more distinct heterogeneous oxidation process at elevated temperatures than in-service conditions.

Other thermoplastics, such as high-density polyethylene (HDPE), are used in energy, industrial and agricultural applications. Chen et al. [7] investigated the aging behavior of HDPE pipelines. They found that pipeline performance diminished as aging time increased, resulting in reduced durability and increased environmental degradation. Other authors, such as Mouallif et al. [8], analyzed the aging effect of HDPE pipes when exposed to sulfuric acid. The results showed a decrease in the elongation and ultimate stress as a function of the increase in temperature and immersion time. In the case of polypropylene (PP), Rjeb et al. [9] examined the impact of natural aging by evaluating three categories of material: unaltered samples, aged samples shielded from light in ambient conditions, and aged samples subjected to both ambient conditions and light. The authors determined via spectral analysis that oxygen exposure led to polymer chain fragmentation. Delbruel et al. [10] examined the weather resistance of three types of PP, i.e., impact-resistant, recycled, and UV-stabilized, which were processed through fused filament fabrication, injection molding, and thermo-compression. The samples were subjected to aging for 6 months under high-hydrothermal and ultraviolet exposures. The results show recycled samples degraded over time, while 3D-printed impact-resistant and UV-stabilized samples resisted aging.

Poly-phenylene sulfide (PPS) is a thermoplastic commonly deployed in a wide range of industries, such as aerospace, automotive, construction, oil and gas, and mining [11]. Yan et al. [12] studied its structure evolution during thermal degradation in nitrogen and oxygen atmospheres using x-ray photoelectron spectroscopy, Fourier transform infra-red spectroscopy and dynamic rheology. The results showed that thermal degradation differed considerably between nitrogen and oxygen environments, with a change in its crystallization behavior monitored using differential scanning calorimetry. Guo and Bradshaw [13] developed an experimental methodology for isothermal aging of PPS films using a dynamic mechanical analyzer. They calculated creep and stress relaxation curves of the material at several temperatures within 15–35 °C below the glass transition temperature at various aging times to determine its isothermal aging behavior. Meanwhile, polyvinylidene fluoride (PVDF) is used in constructing large-span membrane structures owing to its excellent performance, as described by Yang et al. [14]. The authors analyzed diverse mechanical properties, elastic models and aging mechanisms, such as light-induced or high-temperature oxygen aging, to clarify the degradation mechanism of PVDF material over long time exposure.

The degradation mechanisms of thermoplastic materials include plasticization and leaching, among others, revealing that a deep understanding of their mechanical properties after prolonged exposure to high pressure and/or elevated temperatures must inform product design. Traditionally, materials are subjected to severe conditions and extracted from the aging fluids for mechanical evaluation. This method, however, is prone to provide misleading mechanical properties at ambient conditions, particularly for materials where fluid ingress is reversible, which was recently highlighted by scientists at Element Materials Technology Environmental Ltd. [15], Vallourec S.A. and Evonik Industries AG [16]. While performing in situ measurements is an alternative approach, associated techniques are far from trivial as the development of suitable fixtures [17,18] is costly. These challenges have also generated interest in simulation [19] in order to close the knowledge gap.

The American Society for Testing and Materials developed ASTM D638 [20] for determining the tensile properties of unreinforced and reinforced plastics. This standard covers the specifics of sample preparation, testing equipment, and calculations. The test involves subjecting a dog-bone shaped specimen to a controlled tensile load at a specified rate until it deforms or breaks. This allows the determination of tensile strength, elongation at break, yield strength, and modulus of elasticity. ASTM D638 provides a broadly recognized framework for assessing tensile performance used extensively for material characterization, quality control, and performance comparison of different plastic materials. Similarly, ASTM D732 [21] is a standard test method focused solely on determining the shear strength of plastic sheets, plates, or molded shapes. The standard provides detailed requirements for specimen preparation, test apparatus, and calculation procedure although it does not account for edge effects or complex stress states. The test involves applying a shear load under controlled conditions to a flat specimen using a cylindrical punch and die pair, where the punch shears through material with thickness between 1.27 and 12.7 mm; this allows calculating shear strength based on the maximum break load, the specimen’s thickness and the punch’s circumference.

In recent scientific work, shear testing has been correlated to mechanical properties from tensile tests in metal alloys [22] and thermoplastic polymers [23,24,25] but not while samples are exposed to in situ fluids, pressure, and temperature. Empirical relationships have been established to obtain tensile properties from shear test results [17]. In previous publication [1], a modular and compact in situ testing device, an extension of the ASTM D 732 punch–shear method, has been effectively utilized to establish experimental correlations between the tensile and shear properties (yield and modulus) of a PERT sample. These properties were measured at various temperatures using ASTM D638 and ASTM D732 tests under dry conditions. Additionally, the in situ device allowed for the measurement of shear properties in selected polymer samples while immersed in fluids of interest at high temperature and pressure. The present work applies this methodology to various commercially available thermoplastic materials with differing hygroscopicity. The objectives are three-fold, namely: (i) to evaluate the impact of sample preparation methods on measured properties, (ii) to establish correlations between shear and tensile results without fluid exposure, and (iii) to assess the reversibility of measured properties after immersion to saturation in DI water at 95 °C.

## 2. Materials and Methods

Several materials were selected to address the three effects under investigation. An effort was made to acquire, whenever possible, at least two different grades of each polymer type and to include different degrees of hydrophilicity and susceptibility to hydrolysis as part of the selection process. Except for samples meant to study preparation methods and quality control (QC) tracking, all samples were made using an Arburg Centex Allrounder 520C 2000-875 (ARBURG, Loßburg, Germany) commercial injection molding machine. Tensile coupons were injection molded into their final shape (ASTM D638 Type 1) while shear coupons were obtained by water-jet cutting from injection-molded disks of nominal 178 mm diameter and 2.2 mm thickness in a radial pattern to maintain thermal history uniformity. The final dimensions of a shear coupon are 50.8 ± 1 mm outside diameter with a concentric hole of 11 ± 0.5 mm diameter (ASTM D732) and same thickness as the blank. No samples were cut from the central injection point and any parts exhibiting delamination, discoloration, or inclusions were discarded. Other samples were water-jet cut from either extruded or compressed plates to the same final dimensions. The list of all polymers and their codes is shown in Table 1. Note that supplier confidentiality restricts disclosure of more specific details about the thermoplastics investigated in this work.

**Figure 1:**
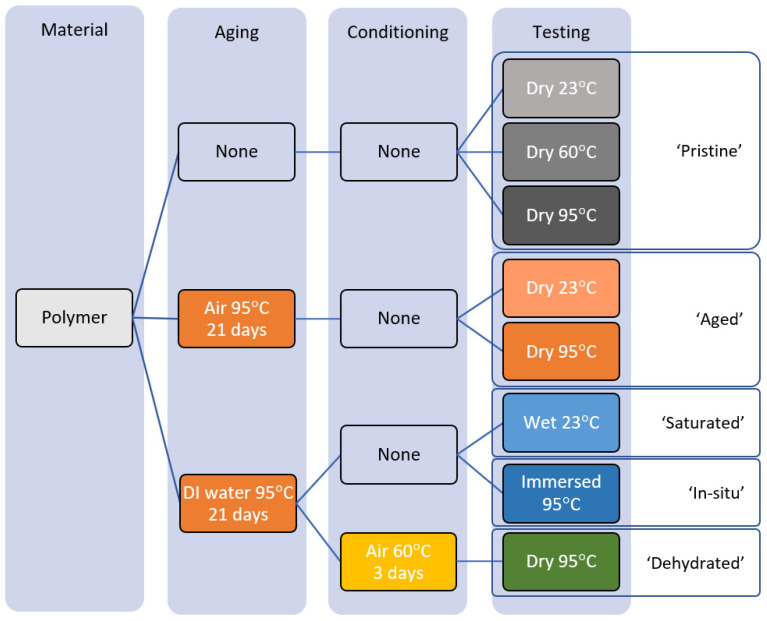
Flowchart summarizing the diverse treatments applied to polymer samples in this study.

A variety of sample treatments were devised to ascertain the effect of thermal aging as well as water exposure (that could lead to saturation and/or hydrolysis) on measured properties. When a sample was measured as received without further thermal treatment, it is referred to as being in a ‘pristine’ condition. An ‘aged’ condition refers to samples that were covered in aluminum foil and stored in a glass jar prior to being subjected to 95 °C for 21 ± 1 days without water exposure, as opposed to ‘saturated’ samples which were segregated by polymer type, held with spacers in a water bath at the same temperature and time conditions. A fraction of the saturated samples was subjected to a drying cycle by holding them at 60 °C for 3 days on wire racks to facilitate air circulation. These samples are referred to as ‘dehydrated’ samples in the present study. For a graphical summary refer to Figure 1.

The weight of saturated samples was monitored at regular intervals using a balance Cgoldenwall Model HZ5003B (Cgoldenwall, Hangzhou, China) after removal from a Delta Design environmental chamber Model MK6300 (Delta Design, LaMesa, CA, USA), drying each coupon and allowing it to equilibrate to room temperature.

Tensile measurements were performed following ASTM D638 [20] using an INSTRON Model 5966 (INSTRON, Norwood, MA, USA) universal testing machine with a 10 kN load cell at a constant rate of 50 mm/min and 50 mm gauge length for all samples. An Epsilon Technology Corp optical extensometer Model ONE-78PT-System (Epsilon ONE, Jackson, WY, USA) was used to measure strain directly and acquired data simultaneously with time, force, stress, and distance channels. ASTM D638 Type 1 samples were tested at constant temperature in pentaplicate either at room temperature (RT, 22–25 °C) or within 3 °C either side of the target temperature inside an oven INSTRON A351-36 (INSTRON, Norwood, MA, USA) after a minimum soak time of 1 h. Coupons were mounted on manual wedge parallel action grips designed to minimize axial preloading with a maximum force rating of 50 kN. The grips were re-tightened at the mid and final point of thermal soak time to prevent slippage. Saturated samples were wiped with a paper towel until no moisture was perceptible on the paper and the measurement started within 1 min to minimize variability in the results.

Shear measurements were performed following ASTM D732 [21] using the INSTRON Model 5966 machine fitted with compression platens in order to distribute evenly the compression load, at a constant rate of 1.25 mm/min for all samples acquiring simultaneous time, force, and distance. Shear coupons were mounted to the punch, tightened via threading with a freely rotating washer to avoid preload and located on the die. This assembly was then inserted into the in situ cell body and clamped firmly in place by a retaining ring. A complete description of this device is given in [1]. Five samples were measured at constant temperature, either at RT or within 5 °C either side of the target temperature inside the body of the device after a 15 min equilibration period. Samples were stored at temperature inside the oven and soaked for at least 1 h prior to measurement. Saturated samples were wiped with a paper towel until no moisture was perceptible on the paper. A slightly different procedure was followed for samples tested ‘in situ’: They are kept in water at 95 °C up to the point of installation, after which water was made to flow through the device until the cavity of the punch was filled, then pressure increased to 1.1 bar to ensure no phase change took place during measurement. The equilibration time was increased to 30 min measured from the point where the water in the cavity was at set-point. To ensure the results comparable to previously published data [1] and to monitor the punch for potential wear, triplicate RT measurements of pristine extruded sheet material (HDPE0, King Performance Commodity High-Density Polyethylene K-Stran—McMaster-Carr catalog number 8619k71) were interspersed and compared throughout the series of experiments, which are referred to as QC samples. For the sake of clarity, Table 2 presents a summary of the measurements performed in this study, as graphically illustrated in Figure 1.

**Figure 2:**
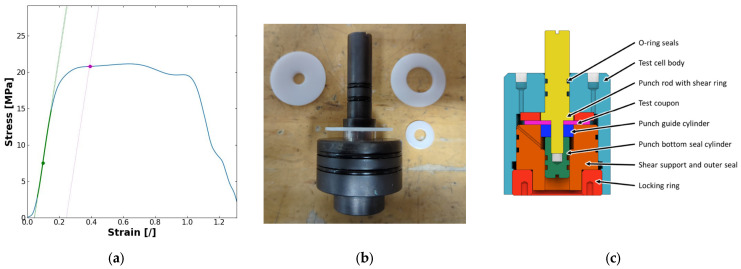
(**a**) Typical stress–strain curve (in blue) obtained from punch–shear experiments demonstrating the elastic region and corresponding linear fit (in green) and the displaced parallel line defining the shear yield value (in magenta); (**b**) photo of a punch–shear rod with sample mounted; coupon before and after testing shown to the left and right, respectively; (**c**) schematic of the in situ punch–shear cell [1].

The tensile modulus was estimated as the slope of the [0.001,0.01] strain region while tensile yield strength was calculated as the point of zero slope on the stress-strain curve as described in [26]. For shear–punch experiments, stress and strain were calculated from the raw data according to the definitions in Equations (1) and (2).

(1) $$\tau =\frac{F}{25.4\pi t}$$

(2) $$\epsilon =\frac{d}{t}$$

where the shear stress τ in MPa is defined as the Force F in N divided by the area of the sheared edge in mm^2^ and strain ϵ as the ratio of distance d in mm to the sample thickness t in mm. The stress–strain curve was then used to estimate the apparent shear modulus as the slope of the linear fit to the elastic region of the stress–strain curve, and shear yield as the point of intersection between the curve and the elastic line displaced positively by 0.2 strain according to the method described in [1] (see Figure 2 for illustration).

Since the mechanical properties of semi-crystalline polymers, including modulus of elasticity and yield stress, are significantly influenced by the degree of crystallinity, as well as the size and distribution of the crystallites [27,28], the PERT materials (PERT PERT1, PERT1a and PERT1b) were characterized for their respective degree of crystallinity using differential scanning calorimetry (DSC Q2000, TA Instruments, Austin, TX, USA). While investigating the effect of crystallinity on mechanical properties is not the focus of the current work, a limited analysis was performed to explain some of the later observations. A sub-sample was cut from each polymer with the average weight of 8 to 10 mg, which were subsequently placed in a hermetic aluminum pan. Nitrogen was selected as the supply gas for DSC tests at a constant flowrate of 50 mL/min. The heating and cooling rates were fixed at 10 °C/min. The sealed assembly was initially subjected to a heat and cool cycle starting from 30 °C to 200 °C. The respective degree of crystallinity χ_C_ was calculated as the ratio of measured sample heat of fusion to that for 100% crystalline polyethylene 293 J/g [29].

## 3. Results and Discussion

### 3.1. Quality Control

All measurements of QC samples were within experimental error at a 95% confidence interval throughout the duration of this study. Hence, it was inferred that no perceptible change in the punch geometry occurred, instilling confidence that the experimental procedure is consistent and repeatable, so this aspect will not be further elaborated on.

### 3.2. Effect of Sample Preparation Method

**Figure 3:**
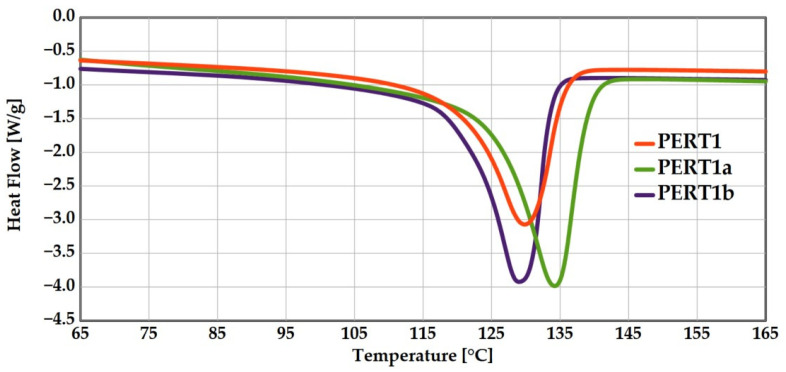
Sample DSC heating scans of PERT1 for the different fabrication methods compared.

A single polymer (PERT1) was selected to test the influence of different preparation methods on the measured thermal and mechanical properties [30,31,32]. The method of fabrication associated thermal history, the measured melting peak temperature (T_M_) and heat of fusion (ΔH_M_) and the calculated degrees of crystallinity (χ_C_) (based on duplicates) are summarized in Table 3. Representative sample DSC heating scans for each type are depicted in Figure 3. As can be seen, the compression molded sample has the highest χ_C_, whereas the injection molded sample has the lowest χ_C_. This finding is plausible, for it has been reported that compression molded polymers generally exhibit a higher degree of crystallinity compared to injection molded polymers due to a slower cooling and lack of shear forces in the compression molding process [33,34].

**Figure 4:**
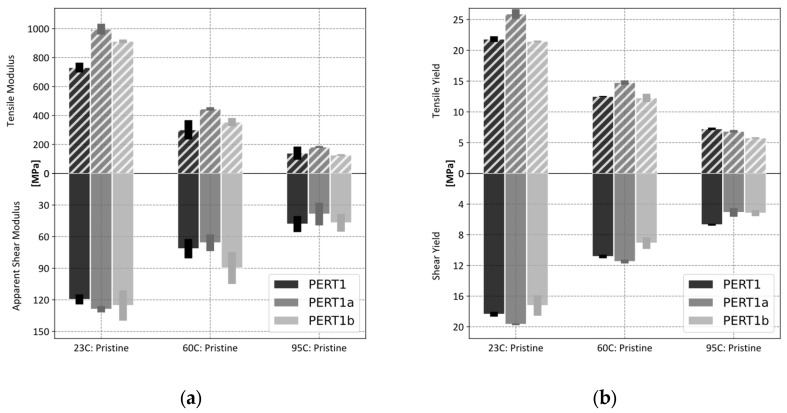
Variation in measured properties as a function of preparation for a PERT polymer; tensile results are shown on the upper half while shear results is shown on the lower portion: (**a**) modulus and (**b**) yield.

As shown in Figure 4, perceptible differences were noted between these samples at 23 °C, with the compression molded samples having a 19% higher tensile yield and 7% higher shear yield; the extruded samples show a 2% lower tensile yield and 6% lower shear yield values when normalized to injection molded samples. The same tendency can be seen in the results obtained at 60 °C, while the results at 95 °C show that values for the compression molded samples (↓5% tensile, ↓24% shear) and extruded values (↓20% tensile, ↓23% shear) drop below the injection molded value. Modulus values obtained from tensile experiments exhibit elevated values for both compression and extruded samples at 23 °C (↑36% and ↓25%, respectively) with a similar trend seen at 60 °C, while at 95 °C compression molded values remain high by 30% and extruded samples drop by 9% as compared to injection molded values. These results are congruous with what has been reported in the literature, i.e., an increase in yield stress as a function of the crystallinity of polyethylene has been demonstrated [30], as well as an increase in tensile strength of compression molded over injection molded samples [31,32]. Apparent shear modulus values show more variability and do not mirror tensile values. Due to the ease of fabrication (i.e., lower fabrication costs) and taking advantage of the fact that injection molded samples bias towards a lower value, it was decided to standardize injection molding as the preferred method for coupon fabrication.

The measured tensile properties (at 23 °C and 60 °C) seem to correlate reasonably well with the measured degree of crystallinity values, see Table 3, following the notion that a higher degree of crystallinity sample yields harder, stiffer, and less ductile behavior [26]. These findings support the fact that the fabrication method and corresponding thermal histories affect the sample crystallinity and, in turn, mechanical properties. Conversely, the shear properties qualitatively conform with the same expected trend only at 23 °C. As will be noted below, the relevant test standard only defines shear yield measurements, and the present study confirms that they serve as a reasonable approximation for the corresponding tensile values. However, interpreting the apparent shear modulus is complex, which will be discussed further in the subsequent text.

### 3.3. Tensile to Shear Correlation

**Figure 5:**
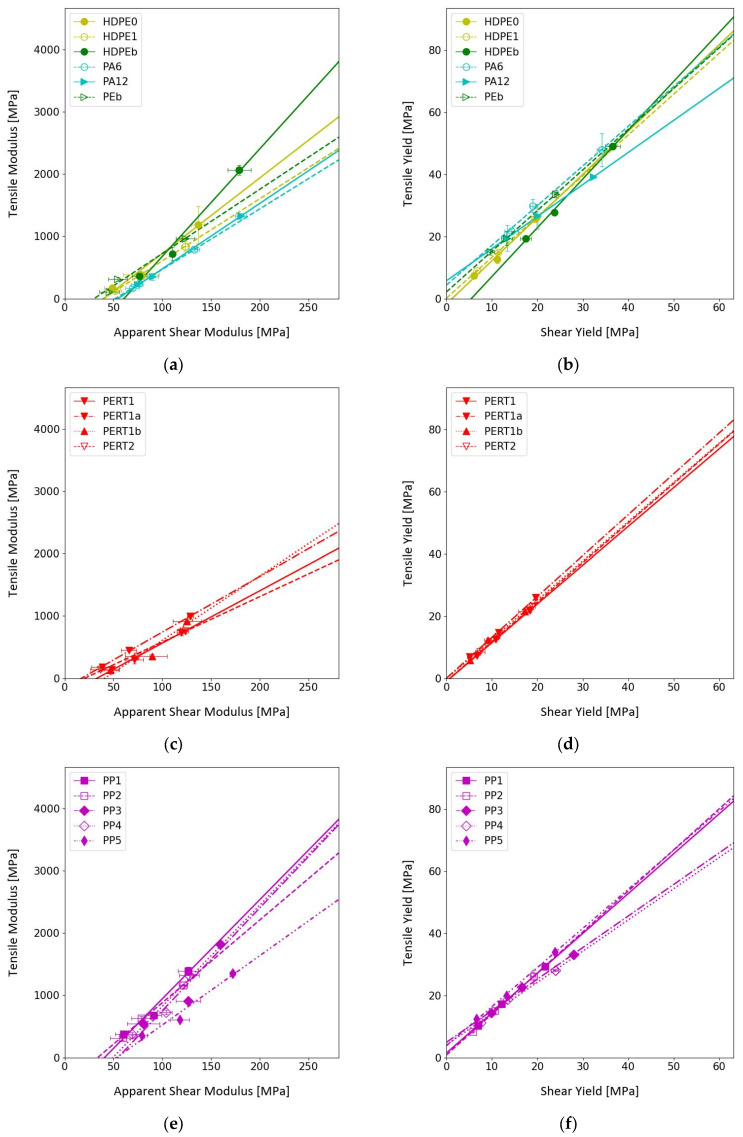

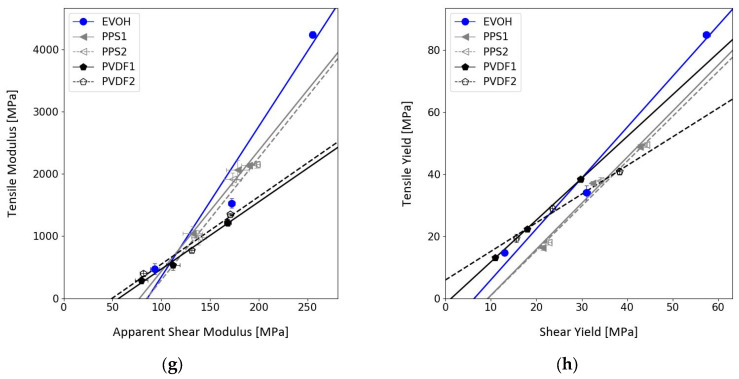
Correlation plots (grouped for clarity). Correspondingly, (**a**,**b**): modulus and yield values for HDPE0, HDPE1, HDPEb, PA6, PA12, and PEb; (**c**,**d**): modulus and yield values for PERT1, PERT1a, PERT1b, and PERT2; (**e**,**f**): modulus and yield values for PP1, PP2, PP3, PP4, and PP5; (**g**,**h**): modulus and yield values for EVOH, PPS1, PPS2, PVDF1, and PVDF2.

In general, it was possible to estimate a linear correlation function between the tensile and shear measurements with just three temperatures (23, 60, and 95 °C). Note that some samples were also measured at 82 °C with no perceptible change on the linear fit, so the effort was concentrated on covering a larger set of polymers rather than collecting data at additional temperatures. The complete list of linear coefficients with their corresponding coefficient of determination (R^2^) can be seen in Table 4. Figure 5 provides graphical summaries for the data presented in Table 4. One can observe that both modulus and yield correlations are linear in this temperature range, making it possible to infer tensile values form their shear counterparts. Furthermore, yield values were on average within 33% of a 1:1 ratio. In contrast, modulus correlations deviate from the ideal correlation by an average factor of 12 while retaining linear behavior.

Referring again to Figure 5 and Table 4, within a given type of polymer, the slopes seem to hold relatively close values. This is clearly illustrated by the PP values: PP1 and PP2 are copolymers sourced from vendor H and their yield correlation values are within 3% of each other; PP3 and PP4 are copolymers sourced from vendor G and their yield correlation values are within 2% of each other while the difference between both groups is on the order of 25%. PP5 is a homopolymer (also sourced from vendor G) that is clearly distinct from the copolymers by approximately 22%.

Greater dispersion in the correlation was noted between the tensile modulus and the apparent shear modulus than between the corresponding yield values. For example, the variability in yield correlation for the three different preparation methods of PERT1 sits at 2%, while the same calculation applied to modulus values is on the order of 19%. From these observations it is interpreted that modulus values exhibit an increased sensitivity to material parameters while remaining linear, which justifies gaining further insight into mechanical property measurement by means of a shear-punch device as a valuable proxy for tensile properties. Notably, while the relevant standard defines only shear yield measurements, this work demonstrates that shear testing may be employed as a reasonable approximation for the corresponding tensile moduli as well. Unfortunately, the interpretation of the apparent shear modulus is not straightforward. The present authors have started an experimental effort to better understand the apparent shear modulus and its meaning, which will be the subject of future research dissemination.

### 3.4. Effect of Evaluation Environment on the Measured Properties

#### 3.4.1. Saturation

**Figure 6:**
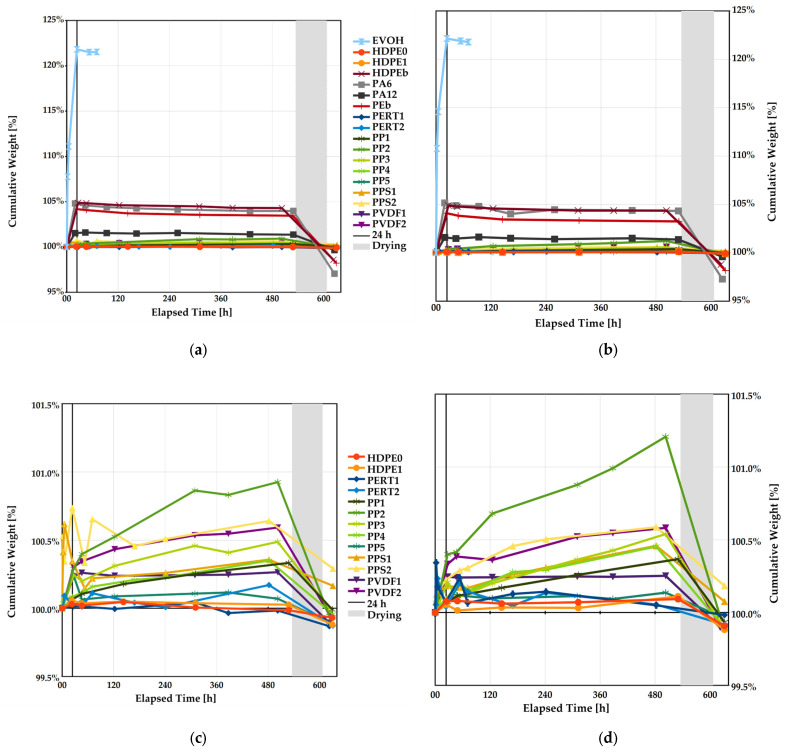
Cumulative water uptake for (**a**) tensile and (**b**) shear test coupons; (**c**,**d**) are expanded views of the lower absorption polymers for tensile and shear test coupons, respectively. The vertical black line marks the first 24 h period, and the shaded area marks the drying period for the dehydrated samples.

Normalized water intake data shows there is no bias with the shape of the coupon and there is general agreement in absorption characteristics. As shown in Figure 6, the polymers can be segregated into four groups based on the absorption behavior, i.e.,

1. EVOH was most affected and became unusable after approximately 72 h;
2. PA and corresponding blends exhibited appreciable absorption, with PA12 being on the lower end;
3. PP copolymers, PPS and PVDF exhibited mild absorption;
4. HDPE, PERT, and PP homopolymer remained mostly unaffected.

#### 3.4.2. Reversibility

**Figure 7:**
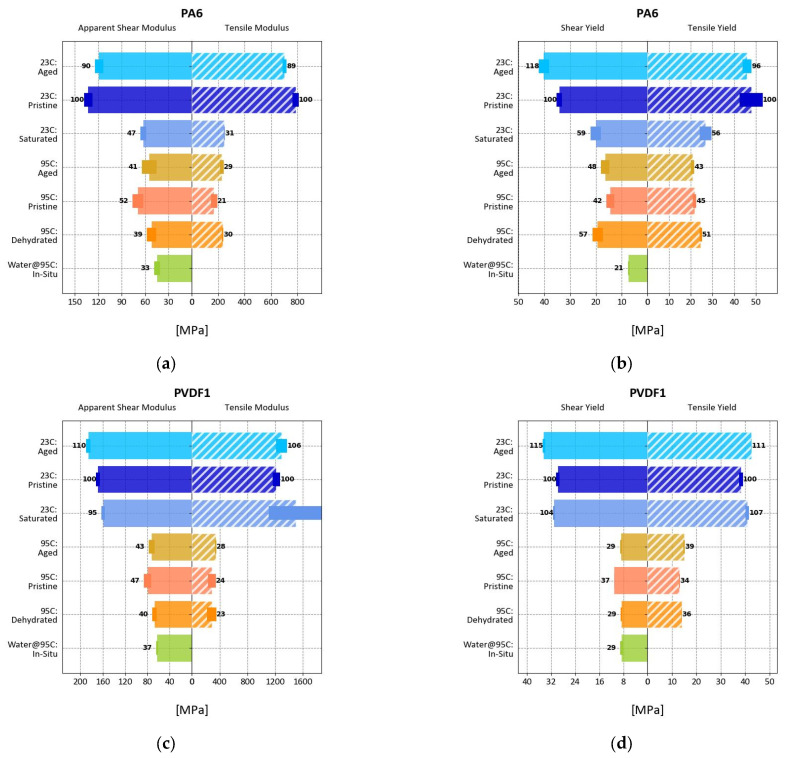
Effect of treatment on the properties of PA6 modulus (**a**) and yield (**b**), and PVDF1 modulus (**c**) and yield (**d**).

To assess the effects of water absorption on the determination of mechanical properties two polymers, sitting in the middle position of their respective classifications, were chosen as examples, i.e., (i) PA6 demonstrates appreciable absorption, and (ii) PVDF1 exhibited minimal but perceptible absorption. Corresponding data are displayed in Figure 7 in a horizontal bar graph to emphasize common measurement conditions. The experimental conditions are labeled along the vertical axis of the graphs indicating the measurement temperature followed by the treatment code. The shear measurement results are shown in solid color bars toward the left-hand side of each panel while the matching tensile results are displayed as hashed bars toward the right-hand side; modulus and yield are shown in their respective panels. The bars originating at the mirrored horizontal axis mark absolute measurements while relative values in percent, normalized to pristine conditions at 23 °C, are designated close to each respective bar.

Focusing on the results obtained at RT, it becomes apparent that both methods (punch–shear and tensile testing) are in general agreement with each other. There is a relatively small change between the pristine and aged samples for both polymers. The saturated samples on the other hand show a significant reduction in value for PA6 and a small change for PVDF1, congruent with water update data.

Results obtained at 95 °C show an overall reduction in value compared to the RT data, which was to be expected. Once again, trends for pristine and aged samples are in general agreement with respect to yield. On the other hand, shear measurements show higher retention of the apparent modulus, which indicates the need for further investigation as already mentioned earlier.

**Figure A1:**
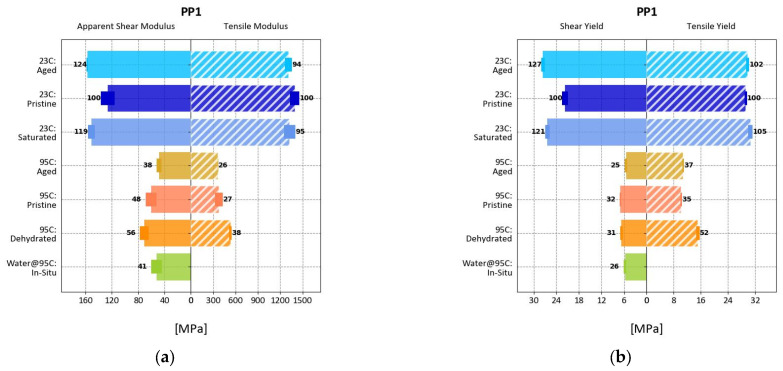

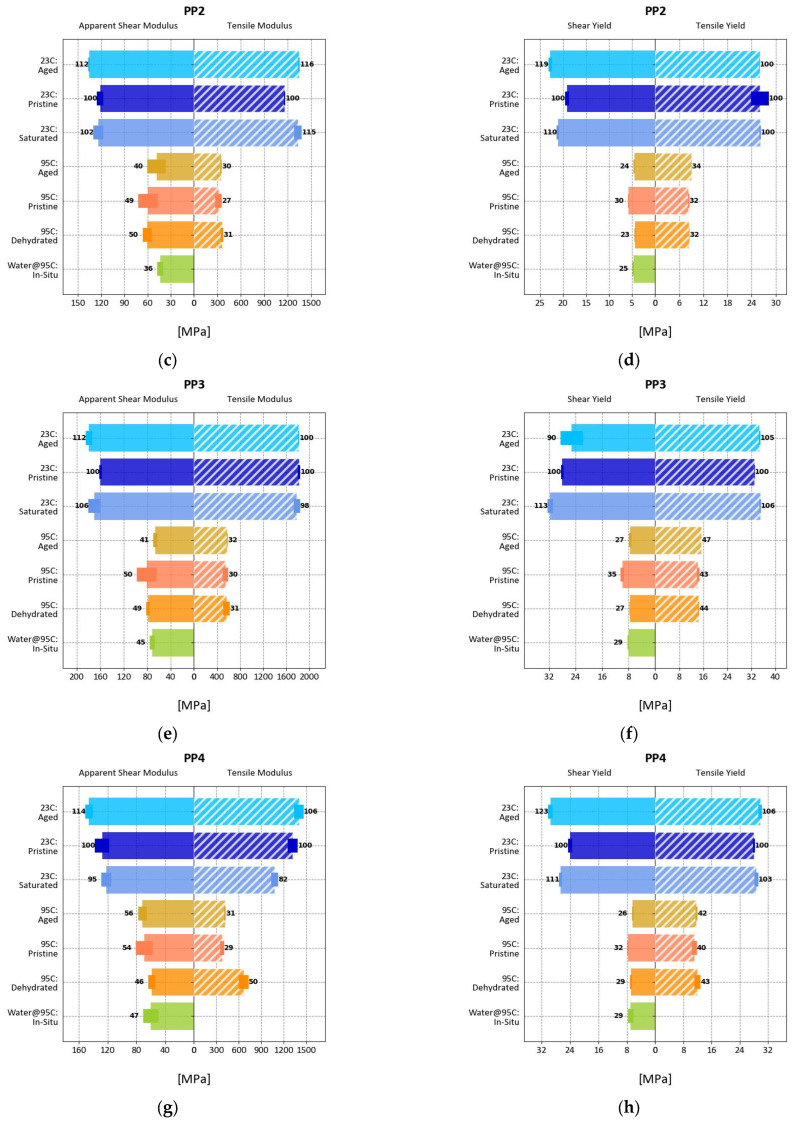

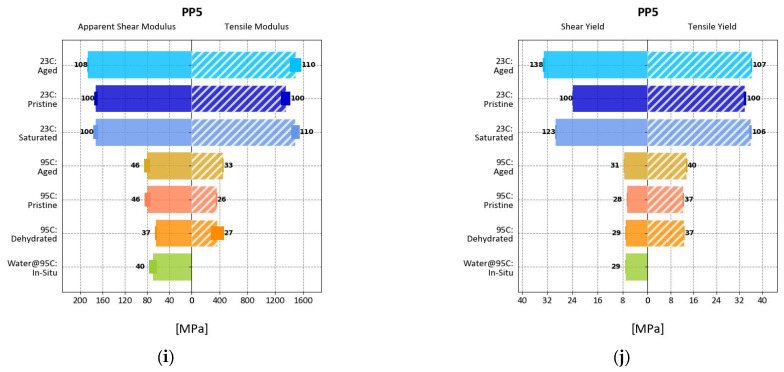
Effect of treatment on the properties of PP1 modulus (**a**) and yield (**b**), PP2 modulus (**c**) and yield (**d**), PP3 modulus (**e**) and yield (**f**), PP4 modulus (**g**) and yield (**h**), and PP5 modulus (**i**) and yield (**j**).

**Figure A2:**
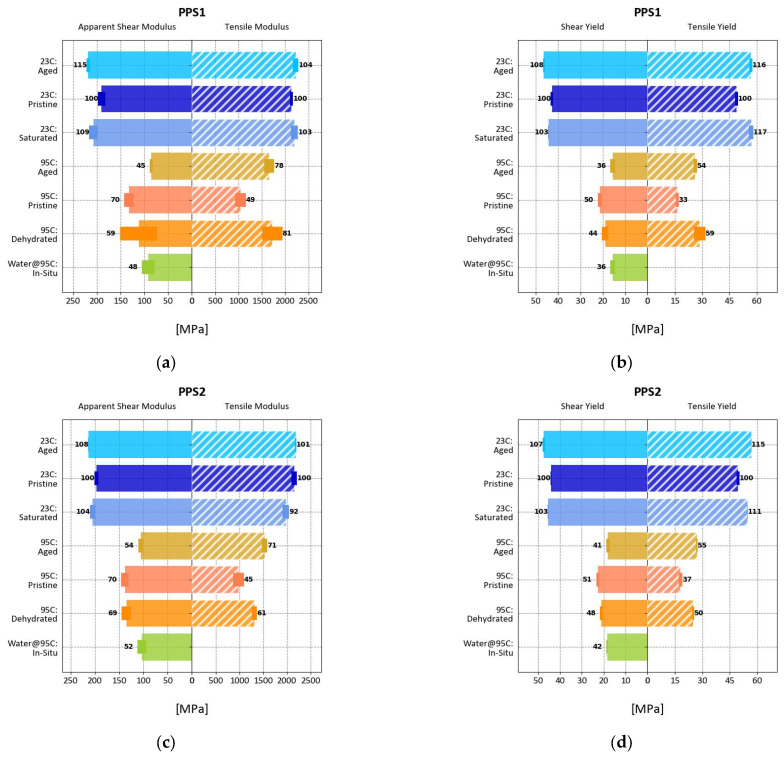
Effect of treatment on the properties of PPS1 modulus (**a**) and yield (**b**), and PPS2 modulus (**c**) and yield (**d**).

**Figure A3:**
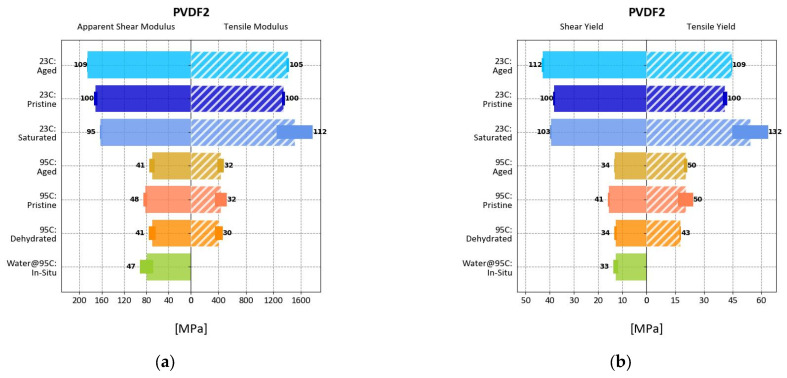
Effect of treatment on the properties of PVDF2 modulus (**a**) and yield (**b**).

**Figure A5:**
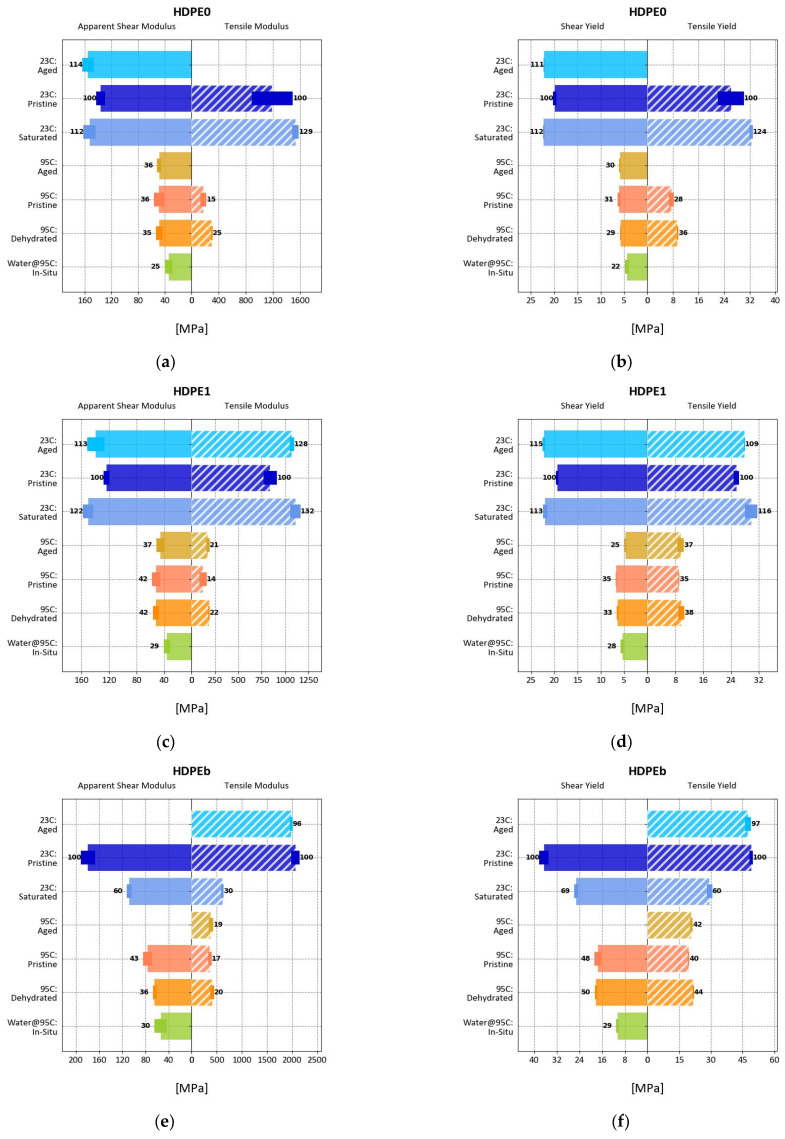
Effect of treatment on the properties of HDPE0 modulus (**a**) and yield (**b**), HDPE1 modulus (**c**) and yield (**d**), and HDPEb modulus (**e**) and yield (**f**).

**Figure A6:**
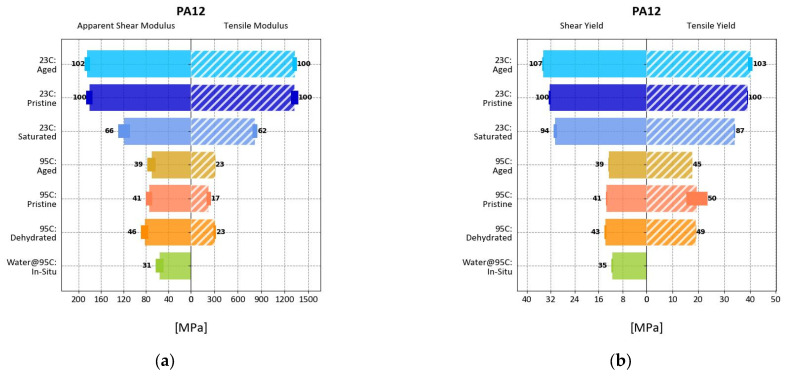
Effect of treatment on the properties of PA12 modulus (**a**) and yield (**b**).

**Figure A7:**
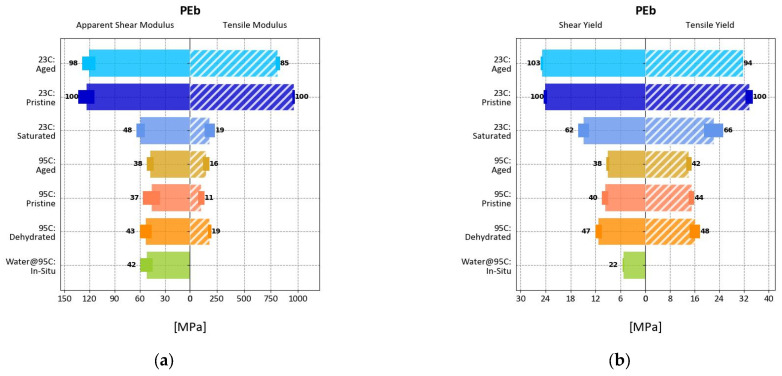
Effect of treatment on the properties of PEb modulus (**a**) and yield (**b**).

For the PVDF1 samples, dehydrated and in situ measurements show no significant difference in values when compared with aged values, as one would expect from water uptake data. Note that similar behavior was observed for PP1, PP2, PP3, PP4, PP5, PPS1, PPS2, and PVDF2, which can be seen in Figure A1, Figure A2 and Figure A3 contained in Appendix A. In contrast, reversibility in properties can be observed for PA6, i.e., the dehydrated values recovered or slightly increased with regard to the pristine sample at 95 °C (from 42% to 57% retention) while the in situ values show a significant reduction (from 42% to 21% retention). This result highlights the importance of in situ testing; relying exclusively on conventional tensile testing, material behavior under service conditions would be overestimated (in as much as in situ testing can replicate them). This effect is also displayed by HDPEb (48~50% pristine/dehydrated to 29% in situ retention), PA12 (41~43% pristine/dehydrated to 35% in situ retention), and PEb (40~47% pristine/dehydrated to 22% in situ retention); the reader is kindly referred to Figure A5, Figure A6 and Figure A7 in Appendix A for further detail.

## 4. Conclusions

The present study explored the experimental correlation between tensile properties (ASTM D638) and shear properties (ASTM D732) of different thermoplastic polymers in pristine conditions, and aged either in dry conditions or after exposure to saturation in deionized water, both at room and elevated temperatures. A suite of commercially available thermoplastic polymers with varying degrees of hygroscopicity was tested. Three major conclusions were drawn from the experimental results.

(i) Using polyethylene with raised temperature as an example, the experiments showed that material fabrication and sample preparation methods can influence the measured properties. Higher crystallinity samples showed increased yield strength values when normalized to injection molded samples.
(ii) For samples in their pristine condition, the study demonstrates a clear correlation between the two test methods for yield strength measurements. However, the interpretation of the apparent shear modulus data from punch–shear testing is not straightforward. While yield correlations are linear and close to a 1:1 ratio (within 33%), the modulus correlations deviate from a 1:1 ratio by average factor of 12 yet retain linear behavior. This observed linearity motivates ongoing efforts by the present researchers to gain further insight into the fundamental aspects that affect apparent modulus measurements.
(iii) The experiments revealed the reversibility of exposure effects after immersion. Therefore, to assess the effects of aging in fluids on the mechanical properties of polymers, in situ punch–shear testing is considered an expedient and superior technique compared to conventional tensile testing, as the latter may overestimate properties. For the polymers that exhibited appreciable absorption, in situ yield strength decreased to approximately half as compared to dehydrated sample values.

The present study demonstrates that in situ shear measurements constitute a valuable tool that allows the estimation of properties not easily accessible with conventional techniques, namely tensile testing. Further work involving aging in aromatic oils and sour conditions is forthcoming.

## Figures and Tables

**Table 1:** List of polymers tested in this study (prepared by injection molding unless specified).

Polymer	Vendor	Note	Polymer	Vendor	Note
EVOH	A	Copolymer	PERT2	G	
HDPE0	B	QC, extrusion	PP1	H	Copolymer
HDPE1	C	Bimodal	PP2	H	Copolymer
HDPEb	D	HDPE-PA blend	PP3	G	Copolymer
PA6	E		PP4	G	Copolymer
PA12	F		PP5	G	Homopolymer
PEb	D	PE-PA blend	PPS1	I	Copolymer
PERT1	C	Bimodal	PPS2	J	Copolymer
PERT1a	C	Bimodal, compression	PVDF1	D	Copolymer
PERT1b	C	Bimodal, extrusion	PVDF2	J	Copolymer

**Table 2:** Synopsis of sample treatments and measurement temperature in °C for all polymers.

Pristine ^1^	Aged ^2^	Saturated ^2^	Dehydrated ^2^	In Situ ^2,3^
23, 60, 95	23, 95	23	95	95

^1^ PERT1, PERT1a, and PERT1b consist of the same polymer but with different preparation methods. ^2^ PERT1a and PERT1b were not included in aging experiments. ^3^ In situ experiments were carried out while the sample was immersed in liquid water at 1.1 bar.

**Table 3:** PERT1 sample fabrication and corresponding degree of crystallinity (χ_C_), melting point (T_M_), and heat of fusion (ΔH_M_).

Polymer	Fabrication Method	T_M_ [°C]	ΔH_M_ [J/g]	χ_C_ [%]
PERT1	Injection molding—air cooled	130	161	55.0
PERT1a	Compression molding—RT cool down in mold	134	186	63.5
PERT1b	Extruded into tape, then cut to shape	129	169	58.0

**Table 4:** Tensile to shear correlation coefficients.

	Modulus ^1^			Yield		
Polymer	Slope [/]	Intercept [MPa]	R^2^	Slope [/]	Intercept [MPa]	R^2^
EVOH	24.22	−2078.92	9.80 × 10^−1^	1.64	−10.47	9.92 × 10^−1^
HDPE0	12.13	−488.20	9.90 × 10^−1^	1.39	−1.77	9.96 × 10^−1^
HDPE1	10	−396.23	1.00 × 10^0^	1.31	0.11	1.00 × 10^0^
HDPEb	17.3	−1054.01	9.94 × 10^−1^	1.57	−8.65	9.99 × 10^−1^
PA6	9.74	−507.09	1.00 × 10^0^	1.28	4.31	9.98 × 10^−1^
PA12	10.52	−574.80	1.00 × 10^0^	1.03	5.7	1.00 × 10^0^
PEb	10.34	−312.89	9.94 × 10^−1^	1.31	2.11	1.00 × 10^0^
PERT1	8.42	−279.11	9.99 × 10^−1^	1.25	−1.06	1.00 × 10^0^
PERT1a	8.94	−154.55	1.00 × 10^0^	1.31	−0.03	1.00 × 10^0^
PERT1b	10.34	−425.47	9.68 × 10^−1^	1.26	−0.13	9.96 × 10^−1^
PERT2	7.3	−149.74	9.96 × 10^−1^	1.27	−0.99	1.00 × 10^0^
PP1	15.95	−653.09	9.86 × 10^−1^	1.28	1.43	1.00 × 10^0^
PP2	13.31	−452.09	9.98 × 10^−1^	1.32	0.78	1.00 × 10^0^
PP3	16.54	−903.66	9.55 × 10^−1^	1.02	4.88	9.97 × 10^−1^
PP4	16.28	−807.27	9.74 × 10^−1^	1	4.07	9.96 × 10^−1^
PP5	11.16	−595.35	9.88 × 10^−1^	1.26	3.75	9.99 × 10^−1^
PPS1	19.45	−1513.70	9.90 × 10^−1^	1.48	−13.90	9.91 × 10^−1^
PPS2	19.7	−1679.49	9.77 × 10^−1^	1.44	−13.59	9.94 × 10^−1^
PVDF1	10.82	−613.17	9.96 × 10^−1^	1.35	−1.77	1.00 × 10^0^
PVDF2	10.88	−540.88	9.86 × 10^−1^	0.92	5.87	9.96 × 10^−1^

^1^ In the case of shear, this refers to the apparent modulus as described in Section 2.

**Figure A4:**
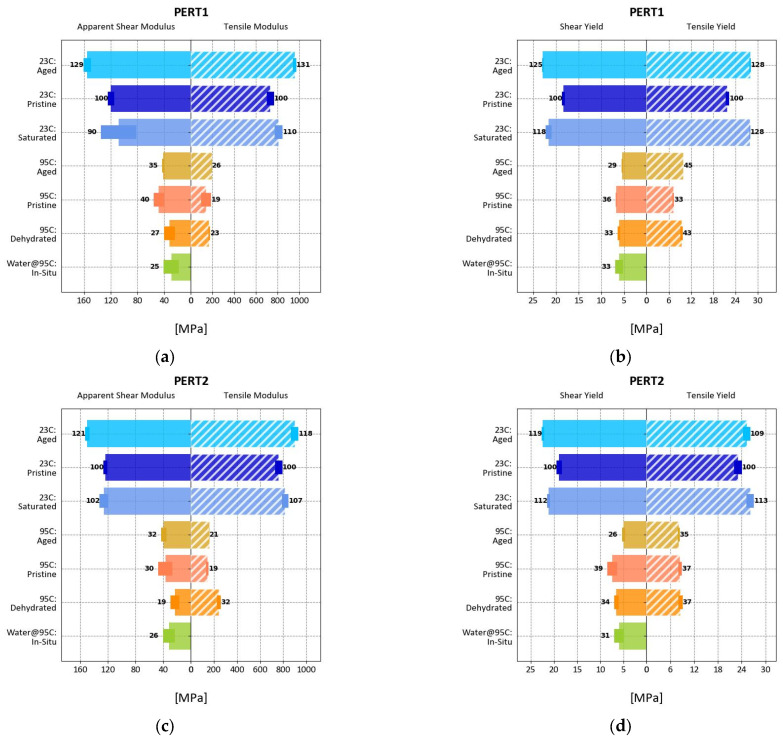
Effect of treatment on the properties of PERT1 modulus (**a**) and yield (**b**), and PERT2 modulus (**c**) and yield (**d**).

## Data Availability

The original contributions presented in this study are included in the article; further inquiries can be directed to the corresponding author.

## Appendix A

Akin to Figure 7, Figure A1, Figure A2, Figure A3, Figure A4, Figure A5, Figure A6 and Figure A7 depicted the test data for the polymers not included in the main body of the text. The graphs follow the format described above. Note that measurements for samples HDPE0 and HDPEb were abbreviated while the EVOH samples were omitted as they were rendered unmeasurable after a few days of aging.
